# Intracardiac echocardiography to guide transseptal catheterization for radiofrequency catheter ablation of left-sided accessory pathways: two case reports

**DOI:** 10.1186/1476-7120-2-20

**Published:** 2004-10-08

**Authors:** Rodolfo Citro, Valentino Ducceschi, Alessandro Salustri, Michele Santoro, Michele Salierno, Giovanni Gregorio

**Affiliations:** 1 Department of Cardiology, "San Luca" Hospital, Vallo della Lucania (Salerno), Italy; 2 Ecocardiography Laboratory, " Portuense" Hospital, Rome, Italy; 3 Boston Scientific Europe Genoa, Italy

## Abstract

Intracardiac echocardiography (ICE) is a useful tool for guiding transseptal puncture during electrophysiological mapping and ablation procedures. Left-sided accessory pathways (LSAP) can be ablated by using two different modalities: retrograde approach through the aortic valve and transseptal approach with puncture of the fossa ovalis. We shall report two cases of LSAP where transcatheter radiofrequency ablation (TCRFA) was firstly attempted via transaortic approach with ineffective results. Subsequently, a transseptal approach under ICE guidance has been performed. During atrial septal puncture ICE was able to locate the needle tip position precisely and provided a clear visualization of the "tenting effect" on the fossa ovalis. ICE allowed a better mapping of the mitral ring and a more effective catheter ablation manipulation and tip contact which resulted in a persistent and complete ablation of the accessory pathway with a shorter time of fluoroscopic exposure. ICE-guided transseptal approach might be a promising modality for TCRFA of LSAP.

## Background

Trans-catheter radiofrequency ablation (TCRFA) has become the treatment of choice for patients suffering from refractory to medical treatment supraventricular tachycardias [1,2]. During percutaneous ablation procedures, catheter location is usually monitored by using fluoroscopy together with the analysis of intracardiac electrograms in order to clarify the mechanisms underlining the arrhythmia and to both locate its origin and record its circuit. This technique may be adequate for several standard ablative procedures, but it has still some limitations concerning the treatment of more complex tachycardia forms.

Left-sided accessory pathway (LSAP) may be ablated using two different modalities: conventional approach through the aortic valve, or transseptal puncture of the fossa ovalis. By using the traditional approach, the left atrium is reached by a retrograde way through the left ventricle and crossing the mitral valve [3,4] whereas, with the transseptal puncture, the mitral ring is reached by an anterograde approach. For this reason, this approach has been considered an alternative and complementary technique for the transvenous introduction of catheters into the left cavities of the heart [5-8]. However, transseptal puncture under fluoroscopic guidance alone, might be hampered by some acute and potentially lethal complications that may be challenging even for expert electrophysiologists [9].

With the technological and miniaturization advances of low frequency transducers capable of enhanced tissue penetration, intracardiac echocardiography (ICE) has become feasible and potentially useful for guiding transseptal puncture and ablation procedures, especially when location of specific anatomic landmarks appears to be more crucial [10,11].

In this manuscript, two cases of ICE-guided catheter ablation of LSAP via transseptal approach have been described.

## Case Presentation

### Case 1

**Figure 1:**
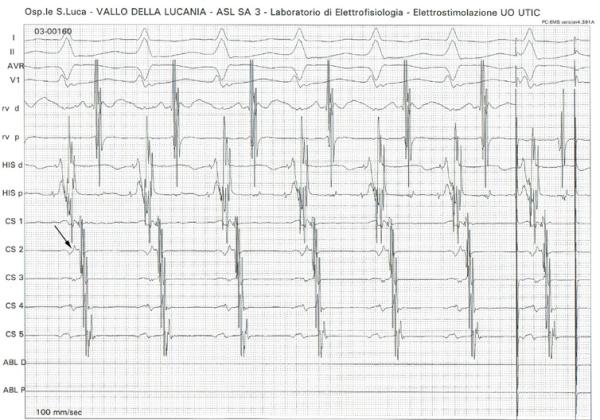
**Orthodromic AV re-entrant tachycardia. **Orthodromic AV re-entrant tachycardia induction with programmed atrial stimulation. Notice VA fusion on CS2 (lateral portion of the mitral ring; see arrow).

**Figure 2:**
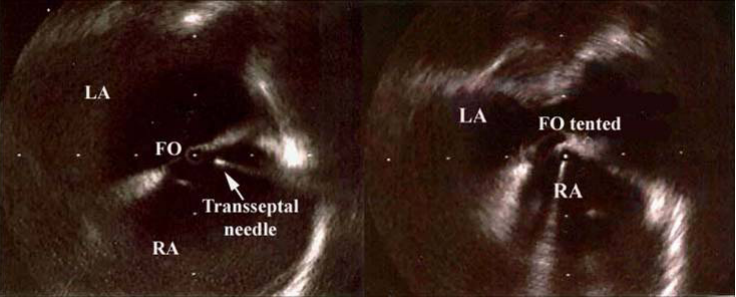
**Brockenbrough needle and the fossa ovalis. **Brockenbrough needle in right atrium approaching the fossa ovalis (right panel); typical "tenting" of the fossa (see arrow) just before septal puncture (left panel). Note the left atrial free wall close to the interatrial septum. (FO = fossa ovalis; LA = left atrium; RA = right atrium).

A 24 year-old woman with Wolf-Parkinson-White (WPW) syndrome and recurrent episodes of sustained supraventricular tachycardia, refractory to medical therapy, was referred to our Department in order to attempt TCRFA procedure. The electrophysiological study was performed off therapy using three diagnostic catheters (one decapolar and two quadripolars) which have been positioned in the coronary sinus (CS1 → CS5 = distal → proximal coronary sinus), His bundle region, and high right atrium/right ventricular apex. The clinical arrhythmia was diagnosed as an orthodromic atrio-ventricular re-entrant tachycardia (AVRT) due to a LSAP that was repetitively induced by both right atrial and ventricular programmed electrical stimulation. The length of the tachycardia cycle ranged from 310 to 300 msec. A well-localized accessory pathway insertion was detected in the lateral left free wall with atrial-ventricular (AV) and ventricular-atrial (VA) intervals of fusion noticeable only in CS2 (Fig 1). During programmed atrial stimulation and AVRT, the transaortic approach was initially attempted by using a 7F-4 mm bidirectional ablation catheter (Blazer HTD, Boston Scientific). Although very short VA and AV intervals (<50 msec without isoelectric line between the two potentials) and satisfactory mean temperature (54–56 C° for 20–30 sec with less than 30 Watts for each radiofrequency application) were obtained, repeated radiofrequency erogations resulted in a transient interruption of the anomalous connection, with recurrence of retrograde conduction along the accessory pathway and atrioventricular re-entrant tachycardia inducibility after a period of 20–30 min. Therefore, by using a mechanical single element ultrasound 9F-9 MHz catheter, an ICE-guided transseptal approach was attempted in order to cranially map the mitral ring (Ultra ICE Boston Scientific/CVIS San José, CA USA). This approach has provided a 360° two-dimensional imaging on a transverse plane perpendicular to the transducer with a radial field of view of approximately 5 cm in depth. ICE catheter was introduced via femoral vein through a 10F long sheath and advanced into the high right atrium. Careful handling of the catheter, along the interatrial septum, provided optimal view of the fossa ovalis that could be easily recognized as a thin area compared to the surrounding atrial structures. The standard approach to transseptal catheterization using a long sheath and dilator over a Brockenbrough needle, was used [5]. The tip of the needle-dilator-sheath apparatus was positioned facing the middle portion of the fossa ovalis. Careful up and down movements of ICE catheter allowed a clear visualization of the needle as well as its contact with the septal wall causing the typical "tenting" effect (Fig 2), which allowed precise location of the puncture of the needle.

**Figure 3:**
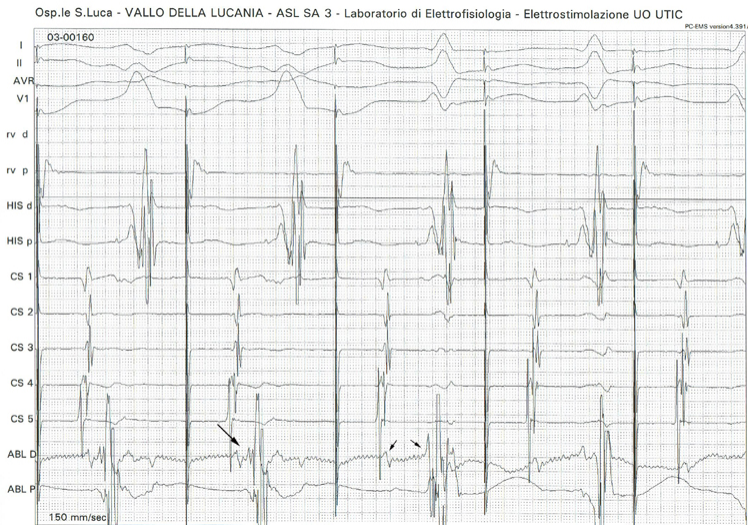
**Ablation of the accessory pathway. **Radiofrequency erogation during atrial pacing with maximal pre-excitation. Notice AV fusion (major arrow) followed by AV split (two minor arrows) on the ABLd recording, corresponding to lateral mitral annulus.

Accessory pathway was completely and definitively ablated with this approach after two erogations lasting each 40 sec. Although the AV and VA intervals that had been selected to deliver radiofrequency energy were similar to those previously recorded, the only difference was a better AV ratio (Fig 3). Such findings might be related to the cranial approach of the atrioventricular ring which has allowed a more stable catheter position on endocardium surface and a lack of lateral sliding, resulting in a higher mean temperature (60° ± 3° vs 54° ± 2°). Radiation exposure was shorter with the transseptal approach (11 vs 19 min) when compared to the retrograde approach.

### Case 2

**Figure 4:**
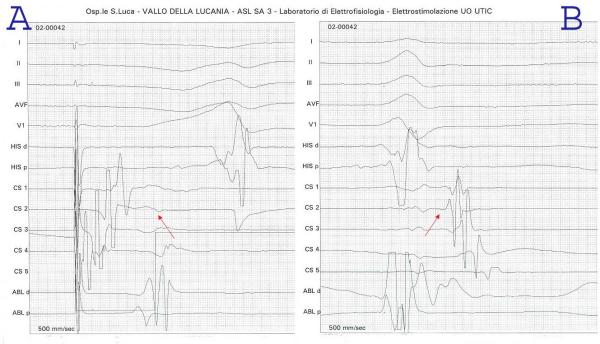
**Left sided accessory pathway. **The shortest AV and VA are recorded in CS2 (see arrows) during programmed atrial stimulation (A) and atrio-ventricular orthodromic re-entrant tachycardia (B) respectively suggesting a left sided posterolateral accessory AV connection.

A 61 year-old man was referred to our Department for a TCRFA procedure due to a recurrent sustained supraventricular tachycardia refractory to antiarrhytmic drugs. The electrophysiological study has been performed by using the previously described protocol. An atrioventricular re-entrant tachycardia, due to an overt LSAP, was repetitively induced by atrial and ventricular programmed electrical stimulation. The length of the tachycardia cycle ranged from 340 to 320 msec. The accessory pathway insertion resulted well-localized in the left lateral mitral ring during AVRT and atrial pacing with maximal pre-excitation (Fig 4A,4B). The trans-aortic approach was firstly attempted by using a 7F- 4 mm bidirectional ablation catheter (Blazer HTD, Boston Scientific). Despite an optimal catheter tip temperature for a reasonable period of time (five erogations reaching a mean temperature of 55° lasting 30–40 sec), only a temporary interruption of the anomalous pathway conduction was obtained. Therefore, ICE-guided transseptal approach was performed as previously described (see additional file: Movie 1 transseptal puncture.avi). The approach resulted in a persistent and complete ablation of the accessory pathway after two successful radiofrequency erogations which have been delivered when the VA interval resulted fused and its ratio was 1,5:1. In this case, ICE allowed a more complete mapping of the mitral ring and a confirmation of effective catheter ablation tip contact. As in the previously described case, the superior approach to the left AV ring resulted in a better manipulation of the ablation catheter in addition to a reduction in fluoroscopic time (10 vs 21 min).

## Conclusion

### Intracardiac echocardiography and catheter ablation procedures

During a standard ICE examination, sequential pull-back of the probe from the superior vena cava through the right atrium to the inferior vena cava allows a detailed identification of important structures such as: the crista terminalis; the right atrial appendage; the caval and the coronary sinus orifices; the fossa ovalis; the ascending aorta and its root; the pulmonary artery; the right ventricle and all of the cardiac valves [12-14]. Thus, this technique has the potential to provide a direct visualization of the endocardium and to precisely locate the ablation catheter which can be identified by the highly specific fan-shaped echocardiographic artefact of the large tip of the ablation electrode [11,12].

ICE is a well-recognized tool to guide ablation procedures. Compared to fluoroscopy, which does not provide definition of endocardial structures, ICE gives a highly accurate evaluation of firm and stable tissue contact. This results in a reduced radiofrequency power output. In addition, it is possible to visualize the lesion site and formation, such as swelling and crater formation. Moreover, possible complications such as occurrence of microbubbles, clot formation and pericardial effusion may be promptly detected through this ultrasound technique [10,15].

A recently developed phased-array intracardiac echocardiography device provides two-dimensional and Doppler images of the heart [10]. Recently, new strategies for ablation of atrial fibrillation (linear atrial ablation and focal ablation of triggers) have been proposed. Phased-Array intracardiac echocardiography enables a direct visualization of the pulmonary veins and allows the assessment of the Doppler flow velocity for all of the pulmonary veins [16]. This technique has been proved to be helpful in monitoring pulmonary vein isolation in patients with atrial fibrillation by improving the outcome and decreasing the incidence of complications such as pulmonary vein stenosis [17].

### Intracardiac echocardiography and transseptal catheterization

Transseptal catheterization is usually performed under fluoroscopic guidance [5]. However, safe transseptal puncture requires detailed visualization of the fossa ovalis that cannot be obtained through fluoroscopy. Thus, it remains a difficult procedure especially for patients which present anatomic abnormalities such as atrial and aortic root dilatation, musculoskeletal disorders and lipomatous hypertrophy of the atrial septum [18]. Furthermore, some complications of the transseptal catheterization due to accidental puncture of adjacent structures [6], such as atrial or aortic perforations, and pericardial tamponade, although rare, can be severe and life threatening. Moreover, in hypovolemic states, the left atrial free wall may be closed to the atrial septum. This potentially dangerous condition can be promptly recognized through the ICE technique, suggesting fluid administration in order to restore an adequate blood volume and to prevent left atrial free wall puncture [19].

The ability to visualize the anatomy of atrial septum and the localization of the fossa ovalis, may greatly enhance both the safety and the efficacy of the transseptal catheterization without any additional morbidity to the procedure [19,20]. As in the above-described cases, ICE allows a continuous monitoring of this procedure showing the tip of the sheath and Brockenbrough needle placed against the middle of the fossa ovalis immediately prior to the puncture [19,20].

Previous reports suggested the use of both transthoracic and transesophageal echocardiography, but these techniques present some limitations [21,22]. The former fails to display, in detail the fossa ovalis, the latter requires sedation which limits communication with the patient during the procedure and increases the risk of hypoventilation.

### Intracardiac echocardiography and left-sided accessory pathway

LSAP represent the majority (59%) of all accessory pathway locations [23]. They can be ablated via transaortic approach. However, severe complications have been reported, including aortic dissection, lesion of the aortic valve, late endocarditis, peripheral and cerebral thromboembolic events [3,4]. Some authors, using the transseptal approach, have reported both a decreased procedural duration and radiation exposure, with higher success rate compared to the transaortic technique [7,24]. Two factors have been taken into account: 1) several left accessory pathways exhibit a broad or oblique insertion on the AV ring, so that they require a multisite radiofrequency energy delivery on both the atrial and ventricular sides in order to be ablated; 2) with the transaortic approach left atrio-ventricular ring mapping becomes a cumbersome procedure due to the retrograde crossing of the ablation catheter through the aortic and the mitral valves. In such a situation, the ablation catheter should be carefully manipulated in order to avoid entrapment into the mitral valve apparatus [4].

The use of ICE during ablation procedures in order to treat LSAP has been previously reported [25]. The observations pointed out by our case report have shown that ICE provides a precise localization of the needle tip position, a clear visualization of the fossa ovalis, a more complete mapping of the mitral ring and a more effective catheter ablation manipulation as well as tip contact. All of these advantages resulted in a persistent and complete ablation of the LSAP with a shorter time in fluoroscopic exposure in opposition to the transaortic approach. It has also been taken into account that a standard TCRFA procedure involves a radiation burden of about 17–25 mSv which appears to be equivalent to what is usually absorbed from natural radiation exposure in during a time period of 10 years and to about 1000 chest X-ray [26]. The combined use of ICE allows to reduce to a half the time of fluoroscopic exposure that induces a significant reduction in radiation load and consequent long term oncogenic risk both for patients and physicians [27].

In conclusion, ICE-guided transseptal approach might be a promising modality for TCRFA of LSAP. However, this report needs to be confirmed by further studies.

## List of abbreviations

AV= atrial-ventricular;

AVRNT= atrio-ventricular re-entrant tachycardia;

CS= coronary sinus;

ICE = intracardiac echocardiography;

LSAP= left sided accessory pathway;

TCRFA= transcatheter radiofrequency ablation;

VA= ventricular-atrial;

WPW= Wolf-Parkinson-White.

## Competing interests

The authors declare that they have no competing interests.

## Authors' contributions

RC carried out intracardiac echocardiography and drafted the manuscript, VD carried out electrophysiological study, AS drafted the manuscript, MS carried out electrophysiological study, MS drafted the revision of the manuscript, GG drafted the manuscript.

All authors have read and approved the final manuscript.

## Supplementary Material

additional file: Movie 1 transseptal puncture.avi Brockenbrough needle immediately after the puncture of the fossa ovalis can be clearly visualized. Click here for file
